# The scope of tobacco cessation randomized controlled trials in low- to middle-income countries: protocol for a scoping review

**DOI:** 10.1186/s13643-020-01361-2

**Published:** 2020-04-21

**Authors:** Navin Kumar, Jessica Ainooson, Ameera Billings, Grace Chen, Lauren Cueto, Kamila Janmohamed, Jeannette Jiang, Raymond Niaura, Amy Zhang

**Affiliations:** 1 Human Nature Lab, Department of Sociology, Yale University, New Haven, CT USA; 2 Department of Social and Behavioral Sciences, College of Global Public Health, New York University, 715 Broadway, New York, NY USA; 3 Department of Epidemiology, College of Global Public Health, New York University, 715 Broadway, New York, NY USA

**Keywords:** Tobacco, Low- to middle-income countries, Cessation, Abstinence

## Abstract

**Background:**

Tobacco consumption is the leading cause of preventable death globally. The global mortality burden of tobacco use lies predominantly in low- to middle-income countries (LMICs). There is much evidence on the effectiveness of tobacco cessation RCTs in high-income nations. However, the evidence base in LMICs is far more limited. To effectively design randomized controlled trials (RCTs) that mitigate tobacco-related harms in LMICs, further understanding of RCTs in this environment will be helpful. We will provide quality evidence regarding the scope of tobacco cessation RCTs in LMICs.

**Methods:**

A scoping review of tobacco cessation RCTs will be conducted. MEDLINE, Embase, PsycINFO, Global Health, Web of Science and Sociological Abstracts will be searched to capture current literature. We will review RCTs that have already been done on tobacco cessation in the LMICs. The key outcome will be tobacco cessation in adults. Examples of the key outcome include smoking abstinence and reduction of tobacco use. Study selection will conform to Preferred Reporting Items for Systematic Reviews and Meta-Analyses Extension for Scoping Reviews (PRISMA-ScR) guidelines and study quality assessed with a modified version of the Cochrane Collaboration’s instrument.

**Discussion:**

As researchers attempt to minimize the harms from tobacco in LMICs, they need to be aware of scientific evidence to develop RCTs to achieve their aim. The review will complement the evidence base on tobacco cessation in LMICs.

## Background

Tobacco consumption is the leading cause of preventable death globally [1, 2]. Most of global mortality burden of tobacco use lies predominantly in low- to middle-income countries [3]. Low- to middle-income countries (LMICs) are experiencing a growing epidemic of tobacco use [4]. Tobacco use rates have been increasing in LMICs [5, 6]. Tobacco control is key to any nation’s public health strategy [7, 8]. Tobacco control, such as cessation interventions, should thus be a priority for policymakers in LMICs to mitigate effects of tobacco-related morbidity and mortality [1, 2].

There are stark differences between LMICs and high-income nations regarding the proportion of smokers who want to quit [9, 10]. Intention to quit smoking in high-income nations is about 75% [11, 12], while LMICs still lag far behind. For example, 41% of Indian smokers and smokeless tobacco users did not want to quit [13]. In high-income nations, prevalence has significantly declined [14]. For example, Australia has seen a 50% decrease in smoking prevalence from 26.1% in 1991 to 13.3% in 2013 [15]. LMIC smoking rates still persist [16].

Previous reviews explored tobacco cessation in LMICs [17–24]. However, these reviews were limited as they did not include studies across all LMICs nor centered on randomized controlled trials (RCTs). There are multiple observational and quasi-experimental studies on tobacco cessation in LMICs [25–27]. However, tobacco cessation RCTs are minimal in this context. While we acknowledge the issues inherent with RCTs [28–30], we also indicate that other study designs are not an adequate replacement for RCTs in establishing efficacy [31], which is key in tobacco cessation [32, 33].

The main objective of this scoping review was to locate and review all published literature relating to tobacco cessation RCTs in LMICs, detailing gaps in the literature. For example, we will detail if there are LMICs where comparatively fewer RCTs have been conducted. We chose to conduct a scoping review due to the broad research question, suited for mapping an area of research [34]. While scoping reviews normally include all evidence, not just RCTs, [35] we sought to focus on RCTs for two reasons. Firstly, previous reviews had centered on other study designs, such as quasi experimental studies. Secondly, to effectively design RCTs that mitigate tobacco-related harms in LMICs, further understanding of RCTs in this environment will be helpful. Research in this area may augment knowledge about tobacco products, broaden understanding around tobacco use among diverse populations, and improve understanding of tobacco firm strategies [36]. Greater understanding around tobacco cessation research may also aid the evidence base to enhance tobacco cessation scholarship, policy, and implementation globally, mitigating the tobacco epidemic [36]. In this scoping review, we will evaluate evidence on the scope of tobacco cessation RCTs, already conducted, in LMICs.

This review will expand on past literature to detail methodological and scientific progress of previously conducted tobacco cessation RCTs in LMICs. We will follow the Preferred Reporting Items for Systematic Reviews and Meta-Analyses Extension for Scoping Reviews (PRISMA-ScR) guidelines, use standard tools to assess study quality [37, 38] and propose a reproducible strategy to query the literature about the scope of tobacco cessation RCTs in LMICs.

## Methods/design

### Search strategy

The search strategy will be performed in line with techniques that enhance methodological transparency and improve the reproducibility of the results and evidence synthesis. Thus, the search strategy will be elaborated and implemented prior to study selection, with the PRISMA-ScR checklist as guidance [37]. We will use the following guiding question to ensure a scoping literature search: “What is the scope of tobacco cessation RCTs in LMICs?”. We will review RCTs already conducted on tobacco cessation in LMICs.

Studies will be reviewed across six databases, including MEDLINE, Embase, PsycINFO, Global Health, Web of Science, and Sociological Abstracts. To account for contemporary studies, a literature search will be conducted till September 2020. No language restrictions will be imposed. Reference lists of the articles will be used to identify more studies. We will also conduct a gray literature search using Google Scholar, clinical trials registries, and governmental websites. We will speak with leading tobacco control experts to identify any relevant studies. EndNote, a bibliographic software, will be used to store, organize, and manage all references [39]. Covidence will be used to manage the title/abstract and full-text screening phases [40]. We will use the search strategy indicated in Additional file 1. We will manually exclude non-RCTs to avoid bias.

### Study selection criteria

Studies will be excluded if they were conducted in the high-income nations. LMICs and high-income nations were defined based on the World Bank’s per capita gross national income metric [41] (see Additional file 2). Two independent reviewers will screen each title and abstract as per inclusion/exclusion criteria (see below). Only studies involving adults ( ≥ 18 years) were included. The key outcome will be tobacco cessation. Examples of the key outcome include smoking abstinence and reduction of tobacco use.

#### Inclusion criteria

- Research was conducted in LMICs
- Research investigating tobacco cessation in adults
- Randomized controlled trials (RCTs)

#### Exclusion criteria

- Any commentaries, editorials, or opinion pieces
- Research conducted in high-income countries
- Qualitative studies
- Non-RCT studies
- Studies involving only children or adolescents

### Study selection

Reviewers will be trained in calibration and utilized standardized screening forms. Reviewers will work in teams of two and independently screen all titles and abstracts that we identify by the literature search strategy. We will obtain full-text articles of all possibly eligible studies and evaluate article eligibility. Reviewers will resolve disagreement around eligibility by discussion or, if necessary, with a third reviewer. Studies reported only as conference abstracts will also be included and we will cite all articles utilizing data from such studies. Conference abstracts are often left out of systematic reviews as they may not contain adequate information [42]. We will include conference abstracts as they are more likely to contain positive results and are often published sooner [42], key to a scoping review on RCTs. We will contact authors where necessary if the abstracts do not provide sufficient information [42].

### Data extraction

Four pairs of reviewers will undergo practice exercises and then work in pairs to independently extract data from studies. Reviewers will resolve disagreement through discussion. When differences are unable to be resolved, a third reviewer will make the final decision. Reviewers will abstract the data using a pretested data extraction template, which includes study design, participants, interventions, comparators, and outcomes.

### Risk of bias assessment

While it is not common to assess risk of bias in scoping reviews [43], we are only including RCTs, and thus, the review is amenable for study quality assessment. Reviewers will work in pairs to independently assess the risk of bias for included RCTs. Disagreements will be resolved by a third reviewer. We will use a modified version of the Cochrane Collaboration’s instrument [44]. The instrument includes nine domains: adequacy of sequence generation, allocation sequence concealment, participant blinding, data collectors blinding, outcome assessment blinding, data analyst blinding, incomplete outcome data, selective outcome reporting, and potential sources of bias. We will contact study authors for additional information when information regarding risk of bias or other methodological aspects is unavailable. The risk of bias will be summarized as a narrative statement and supported by a risk of bias table.

### Descriptive analysis

A narrative synthesis of the outcomes of selected studies will be detailed in the final review. We will include information such as: type of intervention, sample size, target population and demographic characteristics, intervention outcomes.

### Amendments

Any amendments to this protocol will be documented with reference to saved searches and analysis.

### Dissemination

Results of the review will be disseminated in a peer-reviewed journal and likely in other media such as: conferences, seminars, symposia.

## Discussion

The strength of the planned study is the use of a transparent and reproducible procedure for a scoping literature review. In this protocol, we detailed types of studies, participants, interventions, and outcomes included. We stated the data sources, search strategy, data extraction, and quality assessment methods [45]. Through publishing the research protocol, we strengthen the clarity of the search strategy and reduce risk of bias, such as selective outcome reporting [46]. Moreover, we center solely on the scope of tobacco cessation RCTs in LMICs. Results will thus provide high-level information to inform, support, and customize design of RCTs in this setting. As researchers attempt to minimize the harms from tobacco in LMICs, they need to be aware of scientific evidence to develop RCTs to achieve their aim. The planned study hopes to build knowledge around inadvertent outcomes of tobacco cessation interventions and enhance understanding around tobacco control advocacy efforts [36].

## Supplementary information

**Additional file 1.** Medline search example.

**Additional file 2.** Definitions of Low- to middle-income countries (LMICs) and High-Income Nations.

## Data Availability

The datasets used and analyzed are available from the corresponding author on reasonable request.

## Abbreviations

RCT: Randomized controlled trial
PRISMA-ScR: Preferred Reporting Items for Systematic Reviews and Meta-Analyses Extension for Scoping Reviews
LMIC: Low- to middle-income countries
