# Supplementary material for: The scope of tobacco cessation randomized controlled trials in low- to middle-income countries: protocol for a scoping review
Source: Syst Rev. 2020 Apr 21;9:86. doi: 10.1186/s13643-020-01361-2 (PMC7171801; doi:10.1186/s13643-020-01361-2)
Supplement: Supplementary file 1 — Additional file 1. Medline search example. [file 13643_2020_1361_MOESM1_ESM.docx]

Appendix 1

Medline search example

1     (afghanistan or africa or Agalega Island* or algeria or angola or Anguilla or antigua or argentina or Armenia or Armenian or Aruba or Asia or Azerbaijan or bahamas or bahrain or bangladesh or barbados or barbuda or Basutoland or belarus or belize or Belorussia or Belorussian or benin or bhutan or bolivia or borneo or bosnia or botswana or Bouvet Island* or Brasil or brazil or brunei or burkina faso or Burkina Fasso or Burma or burundi or Byelarus or Byelorussian or cabo verde or cambodia or Camerons or Cameroon or Cameroons or cape verde or caribbean or cayman or central african republic or central america or Ceylon or chad or chile or china or Christmas Island* or Cocos Island* or colombia or Comores or Comoro Island* or comoros or congo or Cook Island* or costa rica or cote d'ivoire or cuba or democratic people's republic of korea or djibouti or dominica or dominican republic or dprk or East Timur or ecuador or egypt or el salvador or eritrea or ethiopia or falkland island* or fiji or french guiana or French Polynesia or French Somaliland or gabon or Gabonese Republic or gambia or gaza or Georgia or ghana or Gold Coast or grenada or grenadines or guadeloupe or guam or guatemala or Guiana or guinea or guyana or haiti or Heard Island* or Hercegovina or herzegovina or honduras or Ifni or india or Indian ocean or indochina or indonesia or iran or iraq or ivory coast or jamaica or jordan or Kampuchea or katanga or Kazakh or kazakhstan or Keeling island* or kenya or Khmer Republic or Kirghiz or Kirghizia or Kirgizstan or kiribati or Korea or Kosovo or kuwait or Kyrgyz Republic or kyrgyzstan or Lao PDR or laos or latin america or lebanon or lesotho or liberia or libya or madagascar or Malagasy Republic or malawi or Malay or Malaya or malaysia or maldives or mali or malvinas or marshall island* or martinique or mauritania or mauritius or Mayotte or McDonald Island* or mekong valley or melanesia or mexico or micronesia or middle east or mongolia or montserrat or morocco or mozambique or Muscat or Myanma or myanmar or namibia or nauru or Navigator Island* or near east or nepal or Netherlands Antilles or nevis or new caledonia or New Hebrides or nicaragua or niger or nigeria or Niue or Norfolk Island* or north korea or Northern Mariana Island* or Nyasaland or oman or pakistan or Palau or palestine or Palestinian or panama or papua new guinea or paraguay or peru or Philipines or philippines or Phillipines or Phillippines or pitcairn island* or puerto rico or qatar or reunion or Rhodesia rio muni or Ruanda or rwanda or Sabah or Saint Barthelemy or Saint Helena or saint kitts or saint lucia or Saint Martin or saint vincent or samoa or samoan island* or Sandwich Island* or sao tome or Sarawak or saudi arabia or senegal or seychelles or sierra leone or sikkim or solomon island* or somalia or south africa or south America or sri lanka or St Barthelemy or St Helena or St Kitts or St Lucia or St Martin or St Vincent or sudan or Surinam or suriname or swaziland or syria or syrian arab republic or Tadjikistan or Tadzhik or Tadzhikistan or tajikistan or tanzania or thailand or tibet or timor or tobago or togo or Togolese Republic or Tokelau or tonga or trinidad or tunisia or Turkmen or turkmenistan or "turks and caicos" or Tuvalu or uganda or ukraine or united arab emirates or United Arab Republic or Upper Volta or uruguay or Urundi or Uzbek or uzbekistan or vanuatu or venezuela or viet nam or vietnam or virgin island* or "Wallis and Futuna" or West Bank or West Indies or yemen or Yugoslavia or zaire or zambia or Zimbabwe).hw,ti,ab,cp.

2     Developing Countries.sh,kf.

3     ((southeast or southeastern or western) adj asia).tw,kw.

4     ((developing or less* developed or under developed or underdeveloped or middle income or low* income) adj (economy or economies)).tw,kw.

5     (low* adj (gdp or gnp or gross domestic or gross national)).tw,kw.

6     (low adj3 middle adj3 countr*).tw,kw.

7     (lmic or lmics or third world or lami countr*).tw,kw.

8     transitional countr*.tw,kw.

9     or/1-8

10     exp "tobacco use cessation"/ or exp smoking cessation/ or exp smoking reduction/ or exp harm reduction/ or ((argileh or beedis or betel or chhutta or chillum or cigar* or cigarette* or cigarillo* or dhumti or dokha or e-cigarette* or e-cig* or e-hookah* or gutka or hookah or hookli or imqmik or khaini or kiseru or kizami or makla or midwakh or mishri or mu'assel or narghile or naswar or nicotania or nicotine or paan or pan masala or perique or shisha or smoking or snuff or snus or thoc lao or tobacco or vape or vaping) adj5 (abstinence or cessation or decrease or harm reduc* or harm minimiz* or stop or stopping or withdrawal or quit or quitting)).tw,kw. (49363)

11     9 and 10
