# Supplementary material for: The scope of tobacco cessation randomized controlled trials in low- to middle-income countries: protocol for a scoping review
Source: Syst Rev. 2020 Apr 21;9:86. doi: 10.1186/s13643-020-01361-2 (PMC7171801; doi:10.1186/s13643-020-01361-2)
Supplement: Supplementary file 2 — Additional file 2. Definitions of Low- to middle-income countries (LMICs) and High-Income Nations. [file 13643_2020_1361_MOESM2_ESM.docx]

Appendix 2 – Definitions of Low- to middle-income countries (LMICs) and High-Income Nations

This definition has been adapted from the World Bank and can be accessed at

(<https://datahelpdesk.worldbank.org/knowledgebase/articles/906519>). We defined LMICs as nations falling under the categories below: Low income; Lower-middle income; Upper-middle income.

| **Classification as per World Bank** | **Definition** |
| --- | --- |
| Low-income | Low income economies are those with gross national income (GNI) per capita, calculated using the World Bank Atlas method, of $1,025 or less in 2018 |
| Lower middle-income | Lower-middle-income economies are those with a GNI per capita, calculated using the World Bank Atlas method, of more than $1,026 and $3,995 |
| Upper middle-income | Middle-income economies are those with a GNI per capita, calculated using the World Bank Atlas method, of more than $3,996 and $12,375 |
| High-income | High-income economies are those with a GNI per capita, calculated using the World Bank Atlas method, of $12,736 or more |
